# Molecular Diagnosis of Entecavir Resistance

**Published:** 2010-03-01

**Authors:** Murat Sayan

**Affiliations:** 1 Faculty of Medicine, University of Kocaeli, Kocaeli, Turkey

**Keywords:** Entecavir, Chronic Hepatitis B, Nucleoside Analogue, Entecavir Resistance, Molecular Diagnosis

## Abstract

Entecavir (ETV) is a potent nucleoside analogue against hepatitis B virus (HBV). Because of development of ETV resistance requires at least three amino acid substitutions in HBV polymerase (pol) gene, emergence of ETV resistance is rare (~1%) in nucleoside-naive patients after up to 5 years of treatment. However, it has been suggested that lamivudine (LAM) therapy can preselect for HBV variants associated with resistance to ETV treatment. ETV resistance increased to 51% of patients after 5 years of ETV treatment in LAM refractory patients. The diagnosis of ETV resistance in chronic hepatitis B patients, mainly based on four types of molecular assays: direct sequencing, line probe assay, clonal analysis,and restriction fragment length polymorphism (RFLP) analysis. The applications of other assays are currently more specialized,and their use is more limited. The utility of these assays and their performance characteristics are reviewed below.Briefly, the monitoring of drug-resistant variants is important in the elucidation of the prevalence and mechanisms of resistance development and for the more effective management of treatment options.

## Introduction

In spite of the availability of a highly effective vaccine, approximately one third of the world's population has serological findings of past or present infection with hepatitis B virus (HBV); and globally, over 350 million people are currently chronically infected. The spectrum of disease and the natural history of chronic HBV infection is diverse and variable, ranging from inactive-carrier state to progressive chronic hepatitis, which may evolve to cirrhosis and hepatocellular carcinoma (HCC) [1].Two different types of drugs can be used in the treatment of chronic hepatitis B (CHB): interferon alpha and nucleoside/nucleotide analogues (NUCs). NUCs for HBV therapy belong to three subclasses: L-nucleosides, i.e. lamivudine (LAM), telbivudine (LdT), and emtricitabine (FTC), deoxyguanosine analogues, i.e. entecavir (ETV) and acyclic nucleoside phosphonates, i.e. adefovir (ADV) and tenofovir (TDF). LAM, LdT, ETV, ADV, TDF and FTC have been approved in Europe, the United States, and most Asian and Latin American countries for HBV treatment [1][2].The emergence of mutations should be expect ed from HBV genome characteristics. The major causes of drug resistance include viral factors such as the kinetics of viral production and clearance; lack of a proofreading mechanism during reverse transcription, which creates a large HBV quasispecies pool, and the replication fitness of the viral quasispecies [3][4]. However, a major concern with NUC treatment is the selection of antiviral - resistant mutations. Long-term therapy with NUCs, especially, is associated with an increased risk of the development of drug resistance [5][6]. Mutations selected under NUCs can be split into two groups : those that cause resistance that sometimes leads to a decreased viral fitness, and compensatory mutations, which partially or fully restore the level of viral fitness [7][8].

### Entecavir (Baraclude)

ETV, a novel carbocyclic analogue of 2' - deoxyguanosine, inhibits HBV replication at three different stages:[1] the priming of HBV DNA polymerase, [2] the reverse transcription of the negative-strand HBV DNA from the pregenomic RNA, and [3] the synthesis of the positive-strand HBV DNA. It was approved in 2005 in USA and in 2006 in Europe for naïve and LAM-resistant chronic hepatitis B treatment [9]. ETV has been shown to have more potent antiviral activity than LAM or ADV in head-to-head comparison studies [10][11].

### Entecavir Resistance

ETV resistance was first identified in two patients with LAM-resistant strains, who experienced virologic breakthrough after more than 1 year of ETV therapy [12]. Preliminary data indicate that ETV resistance increased to 51% of LAM-refractory patients after 5 years of ETV treatment [13][14]. More importantly, ETV resistance is rare (~1%) in nucleoside- naive patients after up to 5 years of treatment (Table 1) [15].

Resistance to ETV appears to occur through a two - hit mechanism with initial selection of rt M204V/I mutation followed by amino acid subtitutions at rtT184, rtS202, or rtM250 (Table 1) [12]. Patients who had previous resistance to LAM have lower treatment response and higher resistance rates, because only one or two additional mutations in the HBV polymerase (pol) gene are required for the development of ETV resistance, in contrast to three mutations required in treatment-naive patients. Moreover, LAM-resistance substitutions are necessary for the development of an ETV-resistant mutant [16]. However, some compensatory mutations, such as rtV173L, rtL180M occurring in ETV therapy, help to restore the replication efficiency of the mutant virus [17].

Some specific mutations in the HBV pol region are associated with multidrug failure. A recent example includes rtA181T + rtI233V + rtN236T + rtM250L. It is important to note that rtI233V and rtM250L (in domain E, related to ETV) substitutions do not confer significant drug resistance, nor do they significantly reduce replication capacity in the absence of selection pressure, but appear to act to compensate for the replication defects associated with multidrug resistance [18].

**Table 1:** Cumulative incidence of drug resistant HBV with lamivudine and entecavir [1][7][12][13][14][15][18][39]

	**Lamivudine**	**Entecavir**	**Entecavir**
**Rate of genotypic resistance (year)**	Treatment naive	Treatment naive	Lamivudine resistance
1	24 %	0,2 %	6 %
2	38 %	0,5 %	15 %
3	49 %	1,2 %	36 %
4	67 %	1,2 %	46 %
5	70 %	1,2 %	51 %
**Major mutations conferring resistance**	rtV173L,	rtI169T,	rtL180M + rtM204V/I ± rtI169T ± rtV173L ± rtM250I/V
	rtL180M,	rtT184S/A/I/L/G/C/M,	rtL180M + rtM204V/I ± rtT184S/A/I/L/G/C/M, ± rtS202C/I/G
	rtA181V/T,	rtS202C/I/G,	
	rtM204V/I/S	rtM250I/V (with rtL180M + rtM204V/I)	
**Cross resistance**	Adefovir (rtA181V/T)	Lamivudine (rtM204V/I/S ± rtV173L ± rtL180M)	Lamivudine(rtM204V/I/S ± rtV173L ± rtL180M)
	Telbivudine (rtM204/I)	Telbivudine (rtM204/I)	Telbivudine (rtM204/I)
	Entecavir (intermediate) (rtL180M + rtM204V/I)		
	Emtricitabine (rtV173L, rtL180M, rtM204V/I)	Emtricitabine (rtV173L, rtL180M, rtM204V/I)	Emtricitabine (rtV173L, rtL180M, rtM204V/I)
**Diagnosis of drug resistant HBV**	Direct sequencing	Direct sequencing	Direct sequencing
	Line probe assay^a^	Clonal analysis	Clonal analysis
	Clonal analysis	RFLP analysis	RFLP analysis
	RFLP analysis b		

^a^ INNO- LiPA DR v2 (Innogenetics, Ghent, Belgium)

^b^ RFLP: restriction fragment length polymorphism

### Genotypic Resistance

**Figure 1:**
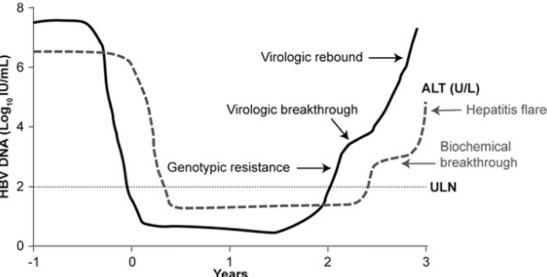
Serial changes in serum HBV DNA and ALT levels in association with emergence of antiviral-resistant HBV mutants. The first manifestation of resistance is the detection of resistance-conferring mutations (ie, genotypic resistance) (5, 20).  ALT: alanine aminotransferase; ULN: upper limit of normal.

Genotypic resistance is generally defined by revelation of viral populations characterized by amino acid substitutions in the HBV pol gene that have been shown to confer resistance to antiviral drugs by in-vitro phenotypic assays [19]. These genotypic mutations generally have occurred in patients who have developed virologic breakthrough, defined as a ≥1 log10 increase in serum HBV DNA above nadir, on two or more occasions 1 month apart while receiving treatment, but genotypic mutations can also emerge in patients with persistent viraemia and without virologic breakthrough (Fig 1)[13]. The testing for genotypic resistance can not be recommended prior to initiation of therapy unless the patient is undergoing treatment for CHB [20]; but, despite advances in HBV genotypic resistance testing, an ETV mutant virus population can be detected at present, in the absence of ETV treatment (in naïve patients and during LAM therapy) [21][22][23][24][25].

### Molecular Assays in the Diagnosis of ETV Resistance

Four types of molecular assays are available for the diagnosis and managment of ETV drug resistance: direct sequencing, line probe assay, clonal analysis, and RFLP analysis. Applications of other assays are currently more specialized, and their use is more limited. The utility of these assays and their performance characteristics are defined in Table 2 . However, the assays more widely used are reviewed below.

**Table 2:** Comparison of the genotyping methods for diagnosis of drug resistance mutation in hepatitis B virus  [6][3][40].

	**Direct sequencing**	**Line probe assay**	**Clonal analysis**	**RFLP**
**Technique**	Population based sequencing	Differential hybridization on the membrane-bound oligonucleotide probe	Cloning of PCR product	PCR and restriction fragment length polymorphism analysis
**Sensitivity**	Affects sequence context and secondary structures in the target, minor subpopulations may escape detection	Minor subpopulations may escape detection	Affects sequence context	Minor subpopulations may escape detection
**Analytic sensitivity**	10% populations of mutants	5% populations of mutants	Can overcome this problem	5% populations of mutants
**Specificity**	Single nucleotide mismatch may escape detection	New set of specific probes are required	Single nucleotide mismatch may escape detection	Must be designed specifically for each mutant of interest
**Mutation detectability**	Detects known and potential new mutations	Only presentsmutations	Detects known and potential new mutations	Only known mutations
**Usability**	Requires sufficient PCR yield and perfect purification (works consistently only for viral loads of over 1E+3 IU/ml)	Requires sufficient PCR yield (works consistently for viral loads over 990 copies/ml (95% CI) a	Requires large number of clones (works consistently only for viral loads of over 1E+3 IU/ml)	Separate sets of endonuclease reactions must be designed (works consistently only for viral loads of over 1E+3 IU/ml)
**Cost**	Expensive but acceptable	Expensive	Expensive	Expensive
**Difficulty**	Time - consuming, requires highly- skilled personnel and expensive equipment	Requires highly- skilled personnel	Time - consuming, requires highly- skilled personnel and expensive equipment	Time - consuming, requires highly- skilled personnel
**Routineness **	Acceptable	Acceptable	unsuitable	unsuitable
**Obtained ing**	In - house	Commercial kit b	In - house	In - house

^a^ 990 copies/ml =1.7E + 2 IU/ml in AmpliPrep/Cobas TaqMan 48 HBV test (Roche Diagnostics GmbH, Mannheim, Germany)

^b^ INNO-LiPA DR v2 (Innogenetfics,Ghent, Belgium).

### Line-Probe Assay

Line-probe assays, using probes for individual mutations, are specific and reproducible and significantly more sensitive in detecting resistance mutations than population-based sequencing. This technology is a useful tool for the rapid and accurate detection of mutants, which make up as little as 5% of the HBV population, with a sensitivity of 990 copies/ml at a 95% confidence interval (CI). However, one disadvantage of the assay (INNO - LiPA DR v2) is the limited scope of the mutations represented in the assay, and it only detects known mutations for LAM and ADV currently with wild type variants [20][22][30]. However, in this technique, recombination of genotypes or new mutations cannot be detected [32]. Periodic updating with new probes specific to novel mutations is required, because HBV strains resistant to newly developed antiviral drugs have been isolated and characterized (Table 2) [33]. On the other hand, an INNO-LiPA DR v3 that confers resistance to ETV therapy has not yet been released as a commercial kit. This prototype line-probe assay allows the detection of variants rtA194T and rtI233V associated with resistance to TDF and ADV, respectively [22].

### Direct Sequencing of the HBV pol Gene Region

The reference method for the detection of resistance-conferring mutations is population-based sequencing (ie, a direct sequence analysis of the HBV pol gene). Genotypic resistance assays use DNA sequencing methods to examine the pol region of the HBV genome for recognizable resistance-associated mutations [26][27][28]. Sequence analysis is considered the gold standard for characterizing HBV DNA isolates [29][30]. However, this assay is time-consuming for a large number of clinical samples, but is suitable for high-throughput screening in a large viral-genome region (Table 2). The in-vitro phenotyping of HBV mutations associated with antiviral resistance in a database program such as geno2pheno (http://coreceptor.bioinf.mpi-inf.) and SEQHEPB tools (http://www.seqvirology.com) (work in the Fasta format of the HBV genomic sequence) is a convenient approach [31]. These tools have great applicability to the interpretation of amino - acid substitutions. The results between amino acid positions 80 - 250 of the HBV pol gene were similarly detected in manual and geno2pheno tool analysis. But, for reliable predictions the sequences must contain the motif "TCCCATCCCATC" starting ateast with codon 159 in the pol frame [24][25].

### Clonal Analysis of HBV Variants

Minor HBV populations can be identified by large - scale cloning and sequencing protocols; however, this is laborious and beyond the capacity of clinical laboratories. This method, however, is sufficiently sensitive for the detection of emerging, resistant mutants that are present in low concentrations [34].For the cloning of the HBV pol region, a 700 bp PCR amplification covering the whole HBV pol gene can be us ed with the following primers; CLC188 5'-TCCCCAACCTCCAATCAC-3' and CLC887 5'-AAACCCAAAAGACCCACAA-3' (designed in our laboratory). The amplified 700 bp HBV pol region can be cloned into a TA vector by using the TOPO-XL-PCR cloning kit (Invitrogen, CA, USA) and the constructs can then be sequenced using the Big Dye Terminator v3.1 Cycle sequencing Kit (Applied Biosystems, Foster City, CA, USA) in an ABI Prism 3130 XL Genetic Analyser (Perkin Elmer, Foster City, CA,USA) according to the manufacturer's instructions [35].

## Conclusions

Other powerful technologies are in development, including high-throughput systems capable of detecting polymorphisms in the entire HBV genome using gene chip technology [36]. Emerging technologies that have not yet penetrated significantly into diagnostic laboratories may become useful in the future.ETV resistance, due to variations at position rt184, rt202, or rt250 alone, seems to have a minor impact on ETV susceptibility [37][38]. However, it should be considered that the addition of rtL180M and rtM204V induces a more than 70-fold decrease in ETV susceptibility; hence, the presence of variants resistant to ETV and LAM may be a factor predictive of ETV- treatment failure.The monitoring of drug-resistant variants is important for the elucidation of the prevalence and the mechanisms of resistance development and for the more effective management of treatment options; and genotypic resistance testing may be tailored to the patient's treatment history and to the severity of the disease.
